# The frequency of SMN gene variants lacking exon 7 and 8 is highly population dependent

**DOI:** 10.1371/journal.pone.0220211

**Published:** 2019-07-24

**Authors:** Raymon Vijzelaar, Reinier Snetselaar, Martijn Clausen, Amanda G. Mason, Marrit Rinsma, Marinka Zegers, Naomi Molleman, Renske Boschloo, Rizkat Yilmaz, Romy Kuilboer, Sylvia Lens, Syamiroh Sulchan, Jan Schouten

**Affiliations:** MRC Holland B.V., Amsterdam, The Netherlands; INSERM, FRANCE

## Abstract

Spinal Muscular Atrophy (SMA) is a disorder characterized by the degeneration of motor neurons in the spinal cord, leading to muscular atrophy. In the majority of cases, SMA is caused by the homozygous absence of the *SMN1* gene. The disease severity of SMA is strongly influenced by the copy number of the closely related *SMN2* gene. In addition, an SMN variant lacking exons 7 and 8 has been reported in 8% and 23% of healthy Swedish and Spanish individuals respectively. We tested 1255 samples from the 1000 Genomes Project using a new version of the multiplex ligation-dependent probe amplification (MLPA) P021 probemix that covers each SMN exon. The SMN variant lacking exons 7 and 8 was present in up to 20% of individuals in several Caucasian populations, while being almost completely absent in various Asian and African populations. This *SMN1/2Δ7–8* variant appears to be derived from an ancient deletion event as the deletion size is identical in 99% of samples tested. The average total copy number of *SMN1*, *SMN2* and the *SMN1/2Δ7–8* variant combined was remarkably comparable in all populations tested, ranging from 3.64 in Asian to 3.75 in African samples.

## Introduction

Spinal Muscular Atrophy (SMA) is one of the most prevalent genetic disorders in Caucasians with an incidence of approximately 1:10.000 newborns. SMA is a recessive disorder with a population dependent carrier frequency ranging from 1:35 in Caucasians to 1:91 in African Americans [1, 2]. Based on age of onset and maximum motor milestones achieved, SMA is divided into different disease categories, distinguished as type 0 to type IV [3–5]. Type 0 and I are the most severe types with SMA onset beginning at birth, patients achieving minimal motor milestones, and a life expectancy of around two years. Intermediate types, type II and III, are milder forms of the disorder where patients are able to sit or walk and most individuals survive beyond 10 years of age. Type IV is the mildest form of SMA with an adult-onset of the disorder and patients having a normal life expectancy [4, 5]. With the recent availability of treatment options, newborn screening and pre-symptomatic treatment of SMA is now being considered in several countries [6].

In more than 95% of SMA cases, the disease is caused by the complete absence of the *SMN1* gene or by *SMN1* to *SMN2* gene conversions [7–11]. In the majority of the remaining cases, intragenic mutations or partial deletions in the *SMN1* gene cause the disease [12, 13]. The copy number of both *SMN1* and *SMN2* is very variable due to instability of the 5q13.2 region, leading to a high frequency of deletion/duplication and gene conversion events [11, 14–17]. In the majority of populations, most individuals have two copies of both *SMN1* and the almost identical *SMN2* gene [18]. There is a single clinically relevant nucleotide difference between *SMN1* and *SMN2*, c.840C>T located in exon 7 [19]. This single nucleotide difference affects splicing of the last coding exon, resulting in 90% of *SMN2* transcripts forming a dysfunctional SMN protein [20, 21]. The complete absence of *SMN2* has no effect on healthy individuals. However, for patients who have no functional *SMN1* gene copies, approximately 10% of SMN functionality is retained per copy of *SMN2*. This results in an inverse correlation between the number of *SMN2* copies and disease severity [8, 13, 22–24].

Both *SMN1* and *SMN2* consist of nine exons, historically named exons 1, 2a, 2b, 3, 4, 5, 6, 7 and 8. Using multiplex ligation-dependent probe amplification (MLPA), the presence of an *SMN1* or *SMN2* gene variant lacking exons 7 and 8 has been reported to have a frequency of 8% in healthy Swedish individuals [18], and 23% in healthy Spanish individuals [25]. During initial validation tests of a new version, B1, of the SALSA MLPA Probemix P021 SMA at MRC Holland the copy number status of both *SMN1* and *SMN2* were determined in a large number of blood derived DNA samples from Dutch individuals. Results showed that approximately 20% of the samples had a lower exon 7 and 8 copy number for the *SMN1* and *SMN2* genes combined as compared to the exon 1–6 copy number. This validation study led us to investigate the prevalence of an SMN variant lacking exons 7–8 in multiple populations. Our study showed a strong inverse correlation between the copy number of SMN gene variants lacking exons 7 and 8 and the *SMN2* copy number, corroborating a report by Alias et al. [26]. This suggests the *SMN1/2Δ7–8* variant in the general population to be predominantly derived from *SMN2* deletion events, in concordance with the difference in deletion frequency between *SMN1* and *SMN2* [24].

**Fig 1:**
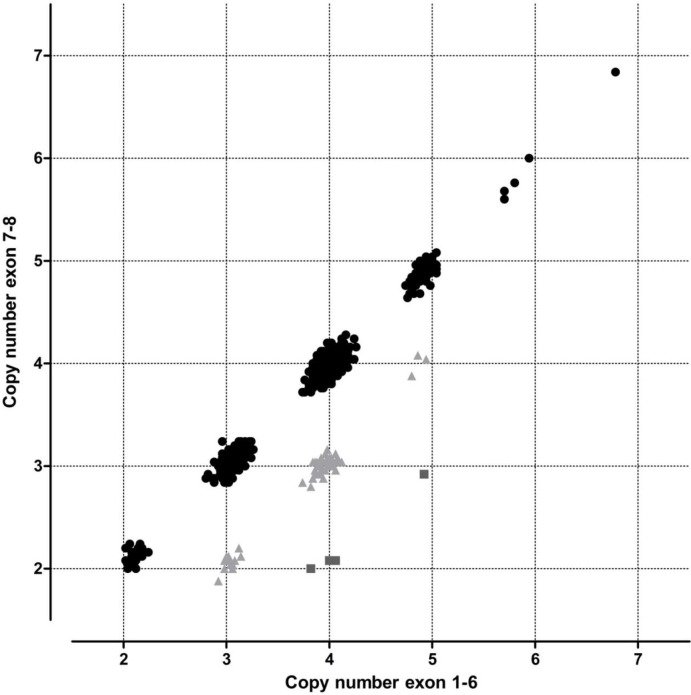
SMN copy number distribution. The distribution of the total copy number of exons 1–6 and exon 7–8 of the *SMN1* and *SMN2* genes combined. *SMN1/2Δ7–8* copies can be identified by a higher copy number of exons 1–6 as compared to exons 7 and 8. Black dots represent samples not carrying *SMN1/2Δ7–8* copies, grey triangles samples containing one copy of *SMN1/2Δ7–8* and dark grey squares samples containing two copies of *SMN1/2Δ7–8*.

In this study 1255 samples from the 1000 Genomes Project comprising of multiple ethnic groups were analyzed using the SALSA MLPA Probemix P021-B1 SMA, which can readily detect the *SMN1/2Δ7–8* variant (Fig 1). The copy numbers for *SMN1*, *SMN2* and *SMN1/2Δ7–8* were determined for each sample and compared for each ethnic group. Our results show that the frequency of the *SMN1/2Δ7–8* variant is strongly population dependent.

## Results

We tested 1255 DNA samples from the 1000 Genomes Project collection with a new B1 version of the SALSA MLPA Probemix P021 SMA. This new P021 probemix has increased gene coverage for the SMN genes as compared to previous versions and covers each exon of the SMN genes with one or more probes. Sequence differences in exon 7 (c.840C>T) and 8 (c.*239G>A) of both *SMN1* and *SMN2* are covered by four probes, in addition 17 probes detect sequences present in both *SMN1* and *SMN2*. Of the latter, ten probes detect sequences in the exon 1–6 region and seven probes detect sequences in the exon 7–8 region.

**Fig 2:**
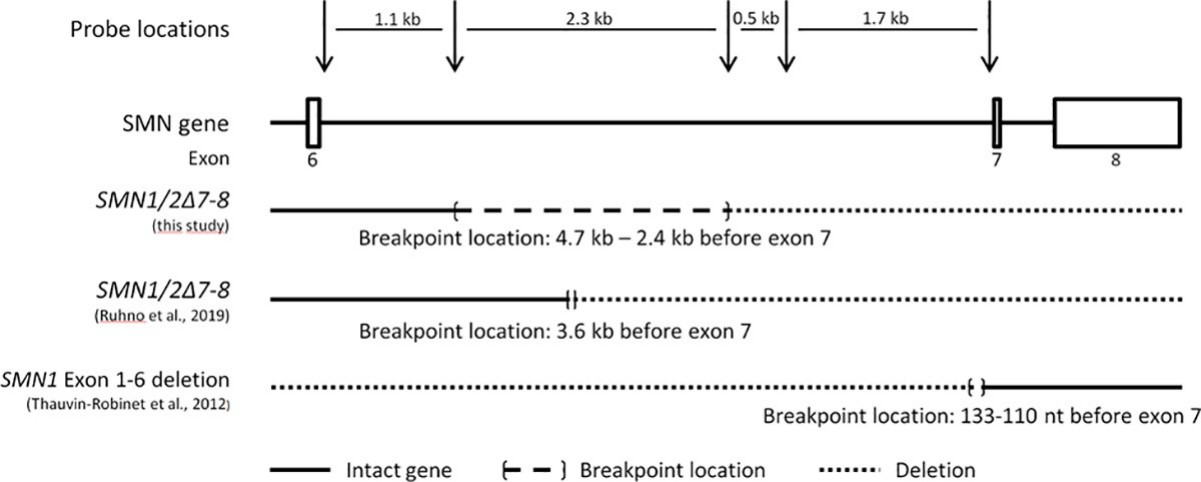
Visualization of both *SMN1/2Δ7–8* and exon 1–6 deletion of *SMN1* breakpoint locations within intron 6. Arrows in the top line indicate the location of the five additional probes designed to map the breakpoint of the SMN variant lacking exons 7 and 8. The third and fourth lines show the breakpoints of the truncated SMN genes.

Upon data analysis, a high frequency of *SMN1/2Δ7–8* variant alleles were observed in specific populations (Table 1), which is in line with previous reports [18, 25]. No other partial gene deletions or duplications were observed. Using synthetic MLPA probes, we located the breakpoint of the *SMN1/2Δ7–8* variant to be in intron 6, between nucleotides 4679 and 2363 before exon 7 (Fig 2). 13 samples were tested and all showed the same probes deviating, indicating that the breakpoint is in the same 2.3 kb region.

**Table 1:** Number of samples containing the *SMN1/2Δ7–8* variant per tested population.

1000 Genomes Project samples	Sample count	One copy *SMN1/2Δ7–8*	Two copies *SMN1/2Δ7–8*	At least one copy *SMN1/2Δ7–8*
Country of origin		Count (%)	Count (%)	Count (%)
Italy	100	18 (18.0)	1 (1.0)	19 (19.0)
Spain	96	13 (13.5)	2 (2.1)	15 (15.6)
England & Scotland	100	19 (19.0)	1 (1.0)	20 (20.0)
Finland	100	8 (8.0)	0	8 (8.0)
**Sum Europe**	**396**	**58 (14.6)**	**4 (1.0)**	**62 (15.7)**
Japan	100	0	0	0
China	100	0	0	0
Bangladesh	104	1 (1.0)	0	1 (1.0)
**Sum Asia**	**304**	**1 (0.3)**	**0**	**1 (0.3)**
Kenya	99	1 (1.0)	0	1 (1.0)
Sierra Leone & Gambia	150	0	0	0
Nigeria	97	0	0	0
**Sum Africa**	**346**	**1 (0.3)**	**0**	**1 (0.3)**
Gujarati Indians	101	7 (6.9)	0	7 (6.9)
Peru & Colombia	108	11 (10.2)	0	11 (10.2)
**Sum Americas**	**209**	**18 (8.6)**	**0**	**18 (8.6)**
**Total samples**	**1255**	**78 (6.2)**	**4 (0.3)**	**82**

Gap-PCR was used to determine if the deletion of exons 7 and 8 of the 82 *SMN1/2Δ7–8* positive samples was of equal size to the exon 7 and 8 deletion described by Ruhno et al [27]. A deletion of equal size was found in 99% of positive samples (81/82). None of 24 negative samples tested positive by this Gap-PCR.

The total number of copies for *SMN1*, *SMN2* and *SMN1/2Δ7–8* in a sample could reliably be quantified by using the median value of the ten MLPA probes for the exon 1–6 region (Fig 1). The total copy number for *SMN1*, *SMN2* and *SMN1/2Δ7–8* ranged from 2 to 7 in our sample cohort. Samples that contained the *SMN1/2Δ7–8* variant could clearly be identified by subtracting the median copy number obtained from the seven MLPA probes detecting exons 7 and 8 of both *SMN1* and *SMN2* from the median copy number obtained from the ten MLPA probes detecting exons 1–6 of both *SMN1* and *SMN2* (Fig 1). In total, we observed 78 samples with one copy and four samples with two copies of the *SMN1/2Δ7–8* gene variant among the 1255 samples tested.

Marked differences in the frequency of the *SMN1/2Δ7–8* gene variant were observed between different populations, as shown in Tables 1 and 2. The frequency of *SMN1/2Δ7–8* copies in the European populations tested was between 8–20%, in line with previously reported frequencies, whereas the number of African and Asian samples with an *SMN1/2Δ7–8* copy was virtually zero (1 in 304 Asian samples, 1 in 346 African samples, 0.3%, Tables 1 and 2). In addition, the average number of either *SMN1* or *SMN2* copies in each individual was found to be strongly population dependent with African populations showing much higher *SMN1* and lower *SMN2* copy numbers in comparison to the other populations examined (Table 2). The number of *SMN1* copies ranged from an average of 2.70 in African samples to 2.03 in European samples. *SMN2* copy numbers were highest in Asian samples with an average of 1.56 and lowest in African samples with an average of 1.05. The total number of SMN copies (*SMN1*, *SMN2* and *SMN1/2Δ7–8* combined) was very similar among the different ethnic populations, ranging from 3.64 in Asian to 3.75 in African samples. However, when excluding the *SMN1/2Δ7–8* copies from the average combined *SMN1* and *SMN2* copy number the European population shows a marked difference, with the average copy number dropping from 3.70 to 3.54 while the other populations remain essentially unchanged (Table 2).

**Table 2:** Average number of SMN copies per population. Asterisks (*) indicate a significant difference in prevalence.

*SMN1* copy number	Europe n = 396 Count (%)		Asia n = 304 Count (%)		Africa n = 346 Count (%)		Americas n = 209 Count (%)	
1	11 (2.8)		2 (0.7)		1 (0.3)		4 (1.9)	
2	364 (91.9)		279 (91.8)		152 (43.9)		177 (84.7)	
3	21 (5.3)		22 (7.2)		143 (41.3)		27 (12.9)	
4	0		1 (0.3)		50 (14.5)		1 (0.5)	
**Average**	**2.03**		**2.07**		**2.70**		**2.12**	
*SMN2* copy number								
0	27 (6.8)		12 (3.9)		80 (23.1)		15 (7.2)	
1	158 (39.9)		117 (38.4)		174 (50.3)		82 (39.2)	
2	195 (49.2)		168 (55.3)		86 (24.9)		107 (51.2)	
3	15 (3.8)		7 (2.3)		6 (1.7)		5 (2.4)	
4	1 (0.3)		0		0		0	
**Average**	**1.51**		**1.56**		**1.05**		**1.49**	
*SMN1/2Δ7–8* copy number								
1	58 (14.6)		1 (0.3)		1 (0.3)		18 (8.6)	
2	4 (1.0)		0		0		0	
**Average**	0.17*		0.003*		0.003*		**0.09**	
Average copy number of *SMN1*,*SMN2* and *SMN1/2Δ7–8*	3.70		3.64		3.75		3.69	
Average copy number of *SMN1* and *SMN2*	3.54		3.63		3.75		3.61	

* p < 0.001

The presence of a *SMN1/2Δ7–8* gene variant is strongly linked to the *SMN2* copy number as shown in Table 3. A significant difference in *SMN1/2Δ7–8* presence was found between individuals carrying two or more copies of either *SMN1* or *SMN2* (p = 1.48e-12, OR: 39.6 [95%CI: 6.9–1573.9]). Presence of *SMN1/2Δ7–8* was nearly absent (0.17%) within the group of two or more *SMN2* copies while more frequently found in carriers of two or more *SMN1* copies (6.73%). When comparing the presence of *SMN1/2Δ7–8* between the groups of one or less copies of either *SMN1* or *SMN2* no significant difference was found (p = 0.39). Samples without *SMN1/2Δ7–8* copies had on average 2.25 *SMN1* copies and 1.44 *SMN2* copies. While samples that contained one or two *SMN1/2Δ7–8* copies had on average 2.02 *SMN1* copies and 0.76 *SMN2* copies. 50.2% of samples (589 out of 1173) without the *SMN1/2Δ7–8* variant had two or more *SMN2* copies, while in the 82 samples that contained one or two *SMN1/2Δ7–8* copies, only one sample had two *SMN2* copies.

**Table 3:** *SMN1* and *SMN2* copy numbers in samples with and without *SMN1/2Δ7–8*.

*SMN1* copy number	All samples Count (%)		Samples without *SMN1/2Δ7–8* copies Count (%)		Samples with one *SMN1/2Δ7–8* copy Count (%)		Samples with two *SMN1/2Δ7–8* copies Count (%)
0	0		0		0		0
1	18 (1.4)		14 (1.2)		2 (2.6)		2 (50)
2	972 (77.5)		900 (76.7)		70 (89.7)		2 (50)
3	215 (17.1)		209 (17.8)		6 (7.7)		0
4	50 (4.0)		50 (4.3)		0		0
Total samples	1255		1173		78		4
**Average *SMN1* copy #**	**2.24**		**2.25**		**2.05**		**1.50**
*SMN2* copy number							
0	134 (10.7)		113 (9.6)		18 (23.1)		3 (75)
1	531 (42.3)		471 (40.2)		59 (75.6)		1 (25)
2	556 (44.3)		555 (47.3)		1 (1.3)		0
3	33 (2.6)		33 (2.8)		0		0
4	1 (0.1)		1 (0.1)		0		0
Total samples	1255		1173		78		4
**Average *SMN2* copy #**	**1.39**		**1.44**		**0.78**		**0.25**

## Discussion

Our results show the frequency of having at least one *SMN1/2Δ7–8* copy to be 15–20% in three European populations (Italian, Spanish, and English/Scottish), which is in line with previous reports [25]. Interestingly, only two out of 650 Asian and African samples tested contained an *SMN1/2Δ7–8* copy, which is approximately 50 times lower than in the tested European populations (Table 1). Furthermore, 99% of 82 *SMN1/2Δ7–8* positive samples had a deletion of equal size as shown by gap-PCR. This strongly suggests that the great majority of *SMN1/2Δ7–8* gene variant copies are the result of an ancient founder event. We noticed an intermediate frequency of *SMN1/2Δ7–8* copies in samples from native North American (Gujarati Indians from Houston; 7%), Peruvian and Colombian (combined 10%), and Finnish individuals (8%).

Our results also confirm previous reports of several African populations having a higher average *SMN1* copy number [1, 2], with an average *SMN1* copy number of 2.70 in African populations and 2.03–2.19 in non-African populations. The increased *SMN1* copy number in Africans is accompanied by a lower average *SMN2* copy number and essentially complete absence of *SMN1/2Δ7–8* copies. Interestingly, our results show that the combined copy number of *SMN1*, *SMN2* and *SMN1/2Δ7–8* is remarkably constant across all populations tested (Table 2), ranging from 3.64 in Asian samples to 3.75 in African samples.

The frequent occurrence of an *SMN1* or *SMN2* variant lacking exons 7–8 was reported first by Arkblad et al in 2006 [18]. Thus far, little attention has been given to this SMN gene variant, likely due to technical difficulties in variant detection [28]. The development of an improved version of the SALSA MLPA Probemix P021 SMA has made the identification and copy number determination of SMN genes, including the *SMN1/2Δ7–8* variant, simple and robust. Our results on 1255 samples from the 1000 Genomes Project show that the presence of this *SMN1/2Δ7–8* variant is almost completely restricted to DNA samples that have an *SMN2* copy number of zero or one (Table 3). Contrary to our results Ruhno et al [27] presented evidence showing that *SMN1/2Δ7–8* can also originate from *SMN1*. This discrepancy could be due to the absence of patients and the small number of SMA carriers within our cohort resulting from the lower frequency of *SMN1* deletion events compared to that of *SMN2* [24]. Only one out of the 590 samples with two *SMN2* copies contained an *SMN1/2Δ7–8* copy, while 81 out of the 665 samples (12%) with zero or only one *SMN2* copy had at least one *SMN1/2Δ7–8* copy. This finding is in agreement with earlier reports [26] where a strong correlation between *SMN2* copy number and the presence of one or two copies of the *SMN1/2Δ7–8* variant was found, further suggesting that most *SMN1/2Δ7–8* variant copies are originally derived from an *SMN2* deletion event.

Using additional MLPA probes, we localized the 3’ breakpoint of *SMN1/2Δ7–8* to intron 6 between nucleotides 4679 and 2363 before exon 7, in 13 samples examined (Fig 2). This is in line with a recent report from Ruhno et al [27] who describe a 6310 bp exon 7–8 deletion that has breakpoints at 3643 nucleotides before exon 7 and 2164 nucleotides after the start of exon 8. The *SMN1/2Δ7–8* breakpoint, located in intron 6, is more than 2 kb from the 3’ breakpoint of a known *SMN1* exon 1–6 deletion that is located between 133 and 110 nucleotides before exon 7 [15]. This latter breakpoint does not contain the recurring repeat sequence identified by Ruhno et al that contains the breakpoint of *SMN1/2Δ7–8*. Thus, the *SMN1/2Δ7–8* copies are not the reciprocal of the recurrent *SMN1* exon 1–6 deletion [15, 18]. It should be noted that the presence of an *SMN1/2Δ7–8* copy could mask an *SMN1* exon 1–6 deletion, although the occurrence would be very rare this could have clinical relevance.

*SMN2* copy number is the most important modifier of SMA disease severity. Next to this, the presence of the c.859G>C variant and/or the intron 6 A-44G, A-549G and C-1897T variants in *SMN2* also seem to be correlated with a milder phenotype [27, 29]. One example of a disease modifier located outside the SMN region is the *PLS3* gene, located on the X chromosome. In siblings, with identical SMN genotypes but discordant phenotypes, the expression level of *PLS3*, is found to influence the severity of the SMA phenotype. High expression levels were found in lymphoblastoid cell lines of asymptomatic but not in symptomatic siblings [30]. The effect of *PLS3* expression is gender specific and not completely penetrant [30, 31] therefore there must be additional disease modifiers inside or outside the SMN region. Several articles have reported healthy adults that lack all *SMN1* exon 7 copies [18, 30, 32–41]. In some cases, the absence of symptoms was attributed to a high *SMN2* copy number (4–5 copies). However, the identification of individuals with homozygous *SMN1* loss and a physique described as “extremely well-muscled” [36] or “athletically built” [32]. As these unaffected individuals had the same SMN2 copy number as their affected siblings, additional disease modifiers outside the SMN genes are expected. The start of SMA newborn screening and pre-symptomatic treatment makes further research on SMA disease modifiers urgent. The *SMN1/2Δ7–8* copy number is unlikely to be a strong disease modifier as its presence was not related to disease severity in the patient cohort studied by Ruhno et al [27]. Besides, Calucho et al. [25] identified 13 SMA patient samples with an *SMN1/2Δ7–8* copy (2%, 13/625), and of these, 11 patients were diagnosed as type I.

The SALSA MLPA Probemix P021-B1 version provides accurate copy number determination of the *SMN1* and *SMN2* genes and the *SMN1/2Δ7–8* variant and will likely result in a more clear picture of the functionality of the *SMN1/2Δ7–8* variant in the future when more SMA patient samples are analyzed.

## Methods

### Subjects

Cell line derived DNA samples from the 1000 Genomes Project [42] were obtained from Coriell laboratories (https://www.coriell.org/). Subsets from the following panels were tested: Bengali in Bangladesh (panel MGP00022); British from England and Scotland (MGP00003); Colombian in Medellin (MGP00005); Esan in Nigeria (MGP00023); Finnish in Finland (MGP00001); Gambian in Western Division-Mandinka (MGP00019); Gujarati Indians in Houston, Texas (MGP00018); Han Chinese South (MGP00002); Iberian populations in Spain (MGP00010); Japanese in Tokyo, Japan (MGP00009); Luhya in Webuye, Kenya (MGP00008); Mende in Sierra Leone (MGP00021); Peruvian in Lima, Peru (MGP00011); Toscani in Italia (MGP00007) and Yoruba in Ibadan, Nigeria (MGP00013). A total of 1255 samples were tested, for which the cell line catalog IDs are listed in S1 Table. The number of samples per subset are shown in Table 1.

### Copy number analysis

A new version, B1, of the SALSA MLPA Probemix P021 SMA was developed at MRC Holland (MRC Holland, Amsterdam, The Netherlands). In contrast to previous P021 versions all exons of the SMN genes are covered by at least one probe in this B1 version. In addition to four *SMN1* and *SMN2* specific probes, each detecting a single nucleotide variation in exon 7 and 8, the probemix contains seven probes that detect a sequence present in, or close to, exon 7 or exon 8 of both *SMN1* and *SMN2*, as well as ten probes that detect sequences present in exons 1–6 of both *SMN1* and *SMN2*.

All MLPA reactions were performed according to the General MLPA protocol (www.mlpa.com). Reactions were analyzed by capillary electrophoresis using the Applied Biosystems 3130 (Life Technologies/Applied Biosystems, Carlsbad, CA). Data analysis was performed using Coffalyser.Net software (www.mlpa.com).

*SMN1* and *SMN2* copy numbers were determined with the use of the *SMN1* and *SMN2* specific probes for exon 7. The *SMN1/2Δ7–8* copy number was determined by subtracting the median copy number obtained for the seven MLPA probes that detect a sequence present in exons 7 and 8 of both *SMN1* and *SMN2* from the median copy number obtained for the ten MLPA probes that detect a sequence present in exons 1–6 of both *SMN1* and *SMN2*.

Five additional probes were designed to investigate the 3’ breakpoint of the *SMN1/2Δ7–8* variant. These probes are specific for sequences located in intron 6 of the SMN genes (Fig 2). Out of the 82 samples containing a *SMN1/2Δ7–8* variant a random subset of 13 samples were tested using these probes.

Hot start gap-PCR was performed in order to determine whether the size of the deletion in *SMN1/2Δ7–8* positive samples is equal to the deletion of 6310 nt described by Ruhno et al [27]. Primers (Forward: /56FAM/CAGTTATCTGACTGTAACACTGTAGGC, Reverse: GTTGTTGCTTATGCTGGTCTTG) were designed based on breakpoints described by Ruhno et al [27] and positive samples generated an amplicon of 583 nucleotides.

### Statistical analysis

Statistics analysis was performed using R version 3.5.1 [43] and Rstudio version 1.1.463 [44]. *SMN1* copy numbers, *SMN2* copy numbers and *SMN1/2Δ7–8* copy numbers were compared using the Fischer’s exact test with the basic R function fisher.test. *SMN1/2Δ7–8* results were dichotomized in a group with either 0 copies or in a group with one or more copies. Presence of the *SMN1/2Δ7–8* was compared within two groups. In the first group *SMN1/2Δ7–8* presence was compared between carriers of one or less copies of either *SMN1* or *SMN2*. In the second group *SMN1/2Δ7–8* presence was compared between individuals carrying two or more copies of either *SMN1* or *SMN2*. Individuals carrying one copy of both *SMN1* and *SMN2* were excluded from the analysis (n = 2). Furthermore, continent data was dichotomized per continent and subsequently compared using the Fischer’s exact test. Bonferroni correction was applied to correct for multiple testing.

## Supporting information

S1 Table SMN copy number distribution and cell line catalog ID. The median copy number value based on both the SMN exon 1–6 probes and SMN exon 7–8 probes is provided for each DNA sample tested, as shown in Fig 1. For each DNA sample, the Coriell Institute calatog ID is provided.
(XLSX)
